# Biosynthesis of Poly(3-hydroxybutyrate-*co*-3-hydroxyhexanoate) from CO_2_ by a Recombinant *Cupriavidus* *necator*

**DOI:** 10.3390/bioengineering8110179

**Published:** 2021-11-07

**Authors:** Kenji Tanaka, Kazumasa Yoshida, Izumi Orita, Toshiaki Fukui

**Affiliations:** 1 Department of Biological and Environmental Chemistry, Faculty of Humanity-Oriented Science and Engineering, Kindai University, Fukuoka 820-8555, Japan; 2133950002m@ed.fuk.kindai.ac.jp; 2 School of Life Science and Technology, Tokyo Institute of Technology, Yokohama 226-8501, Japan; iorita@bio.titech.ac.jp (I.O.); tfukui@bio.titech.ac.jp (T.F.)

**Keywords:** biodegradable plastic, PHBHHx, CO_2_, *Cupriavidus necator*, hydrogen-oxidizing bacterium

## Abstract

The copolyester of 3-hydroxybutyrate (3HB) and 3-hydoxyhexanoate (3HHx), PHBHHx, is one of the most practical kind of bacterial polyhydroxyalkanoates due to its high flexibility and marine biodegradability. PHBHHx is usually produced from vegetable oils or fatty acids through β-oxidation, whereas biosynthesis from sugars has been achieved by recombinant strains of hydrogen-oxidizing bacterium *Cupriavidus necator*. This study investigated the biosynthesis of PHBHHx from CO_2_ as the sole carbon source by engineered *C. necator* strains. The recombinant strains capable of synthesizing PHBHHx from fructose were cultivated in a flask using complete mineral medium and a substrate gas mixture (H_2_/O_2_/CO_2_ = 8:1:1). The results of GC and ^1^H NMR analyses indicated that the recombinants of *C. necator* synthesized PHBHHx from CO_2_ with high cellular content. When 1.0 g/L (NH_4_)_2_SO_4_ was used as a nitrogen source, the 3HHx composition of PHBHHx in the strain MF01∆B1/pBBP-ccr_Me_J4a-emd was 47.7 ± 6.2 mol%. Further investigation demonstrated that the PHA composition can be regulated by using (*R*)-enoyl-CoA hydratase (PhaJ) with different substrate specificity. The composition of 3HHx in PHBHHx was controlled to about 11 mol%, suitable for practical applications, and high cellular content was kept in the strains transformed with pBPP-ccr_Me_J_Ac_-emd harboring short-chain-length-specific PhaJ.

______________________________________________________________________________

## 1. Introduction

Plastic wastes reaching marine environments fragmented into microplastics, but they cannot be biologically assimilated, and, unfortunately, many biodegradable plastics are also not decomposed in marine environments [1]. In contrast, bacterial polyhydroxyalkanoates (PHAs) have been shown to be biodegraded in marine environments [2]; thus, they are attracted as eco-friendly biodegradable plastics that are alternatives to petrochemical-derived plastics. More than 150 kinds of hydroxyalkanoate units have been identified as constituents of PHAs [3]. *Cupriavidus necator* H16 is the bacterium that has been used the most and studied for the biosynthesis of PHAs because it has a high ability to accumulate PHAs. *C. necator* can assimilate many types of organic compounds in heterotrophic culture conditions to accumulate PHAs within the cells. 

A homopolyester of (*R*)-3-hydroxybutyric acid, P(3HB) is well known and the best studied PHA. However, P(3HB) is stiff and has relatively poor in impact strength [4]. Melting and thermal degradation temperatures of P(3HB) are 170–180 °C and 180–190 °C, respectively, resulting in very narrow processability windows caused by low resistance to thermal degradation [5]. A copolyester of 3HB and (*R*)-3-hydroxyhexanoic acid (3HHx), P(3HB-*co*-3HHx) [PHBHHx] is very attractive as it has high flexibility and properties that are similar to several common petroleum-based plastics. Additionally, PHBHHx shows excellent biodegradability even in seawater [6]. Since PHBHHx was firstly found in *Aeromonas caviae* FA440 in 1993, many researchers have been studied for PHBHHx and its microbial synthesis. Kaneka Corporation (Tokyo, Japan) has constructed a PHBHHx (Kaneka Biodegradable Polymer Green Planet™ (PHBH™)) plant of which its production capacity was around 5000 tons per annum in 2019 [7]. It is known that 3HHx composition is an important factor for thermal and mechanical properties of PHBHHx. The melting temperature (*T_m_*) of PHBHHx decreases according to the increase in 3HHx composition [5]. For instance, *T_m_* of P(3HB-*co*-10mol% 3HHx) synthesized from olive oil by *A. caviae* is 136 °C, and break elongation of the copolymer is about 400% [8]. Unfortunately, the accumulation of PHBHHx in *A. caviae* is not so high, as the polymer content in the cells is within the range from 20 to 30 wt% [9]. Meanwhile, biosynthesis of many PHA copolymers usually requires the addition of a precursor compound structurally related to a second unit other than 3HB. Such precursor compounds are usually expensive and often toxic. The efficient production of useful PHA copolymers from inexpensive feedstocks is an urgent task needed for wider use in society [10]. Genetic modification of PHA-producing microbes allows us to modify or construct pathways for biosynthesis of desired PHAs from various kinds of carbon sources. There have been many reports for the biosynthesis of 3HB-based copolymers from plant oils or fatty acids, where (*R*)-3HA-CoAs are provided via β-oxidation of acyl-moieties. We have further constructed *C. necator* H16-based strains capable of synthesizing PHBHHx from structurally unrelated sugars [11]. An artificial pathway was designed for the generation of (*R*)-3HHx-CoA from sugar-derived acetyl-CoA molecules and installed into *C. necator*. One of the strains, MF01ΔB1/pBPP-ccr_Me_J4a-emd, accumulated P(3HB-*co*-22mol% 3HHx) in the cells with high cellular content on fructose.

*C. necator* is a facultative hydrogen-oxidizing bacterium that can also grow chemolithoautotrophically by using CO_2_ as the sole carbon source and H_2_ and O_2_ as energy sources. The former name of *C. necator* was *Alcaligenes eutropha*, *Hydrogenomonas eutrophus*, *Wautersia eutropha* and *Ralstonia eutropha*. Hydrogen-oxidizing bacteria are known to glow rapidly with high cell yields on CO_2_ owing to their high CO_2_-fixation ability, of which its level is the highest among all autotrophic organisms. It is, therefore, beneficial to use hydrogen-oxidizing bacteria for industrial bioprocesses in order to convert CO_2_ to new cellular materials [12] as well as “single cell protein,” of which its amino acid profile is similar to high-quality animal protein [13]. *C. necator* is also one of the best suitable species for the production of the marine biodegradable plastic PHAs by using CO_2_ as a carbon source. We have already succeeded in the production of P(3HB) from CO_2_ by autotrophic high cell density cultivation of *C. necator* and other hydrogen-oxidizing bacteria [14,15,16,17,18]. 

In this article, we report the biosynthesis of PHBHHx from CO_2_ by the two engineered strains of *C. necator* H16 and by further modified strains to synthesize PHBHHx with 3HHx composition suitable for practical applications, as well as the effects of the introduced genes on the synthesis and composition of the copolymer.

## 2. Materials and Methods

### 2.1. Construction of Plasmids and Strains

The bacterial strains and plasmids used in this study are listed in Table 1. The details for the construction of recombinant plasmids and strains were described in our previous report [11,12,13,14,15,16,17,18,19,20]. The genetic modifications in *C. necator* H16 (wild type) are summarized as follows. In strain MF01, the short chain length (scl)-specific β-ketothiolase gene *phaA* and PHA synthase gene *phaC* in *pha* operon on chromosome 1 were replaced with *bktB* and *phaC*_NSDG_, respectively [20]. *bktB*, encoding medium-chain-length (mcl)-specific β-ketothiolase, is originally located at the inherent locus (h16_A1445) on the *C. necator* chromosome, and the second copy was used to replace *phaA* in the *pha* operon. *phaC*_NSDG_ encodes the N149S/D171G double mutant of PHA synthase, possessing broad substrate specificity from C_4_ to C_7_ derived from *A. caviae* [21]. MF01∆B1 was constructed by further deletion of the NADPH-dependent acetoacetyl-CoA reductase gene phaB1 in strain MF01 [11]. PhaB1 is the major reductase for the conversion of acetoacetyl-CosA to (*R*)-3HB-CoA, and *C. necator* has the minor homolog PhaB3 that partially contributes to P(3HB) biosynthesis. In strain MF01∆B1, deletion of *phaB1* was supposed to change metabolic flux distribution at acetoacetyl-CoA node, resulting in the enhanced formation of crotonyl-CoA via (*S*)-3HB-CoA [20]. pBPP-ccr_Me_J4a-emd [11] has been constructed by inserting *ccr_Me_* (crotonyl-CoA carboxylase/reductase gene from *Methylorubrum extorquens*), *phaJ4a* (mcl-specific (*R*)-enoyl-CoA hydratase gene from *C. necator* [22]) and *emd_Mm_* (a codon-optimized gene encoding ethylmalonyl-CoA decarboxylase derived from *Mus musculus*) at the site downstream of the *phaP1* promoter in the broad-host-range expression vector pBPP [23]. Another plasmid pBPP-ccr_Me_J_Ac_-emd was constructed by replacing *phaJ4a* with a scl-specific (*R*)-enoyl-CoA hydratase gene *phaJ_AC_* derived from *A. caviae* [20]. Either plasmids were introduced into strains MF01 or MF01ΔB1 by transconjugation using *E. coli* S17-1. These four recombinant strains were tested for the biosynthesis of PHBHHx from CO_2_ in flask culture under autotrophic conditions.

### 2.2. Culture Medium and Condition

**Figure 1:**
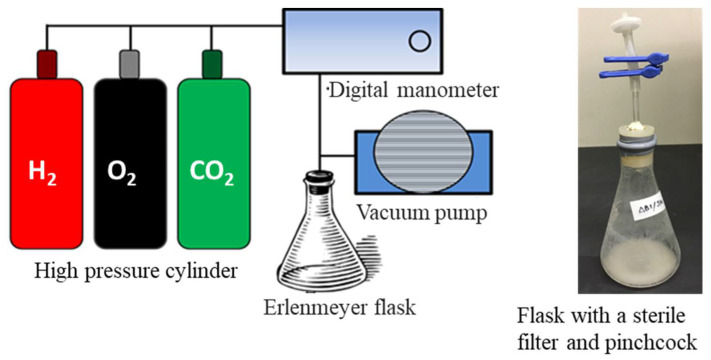
Apparatus for flask culture of *C. necator* in autotrophic condition and supplying substrate gas mixture.

A complete mineral salts medium was used for the autotrophic culture of the recombinant strains of *C. necator.* The basic composition of the mineral salts medium was (NH_4_)_2_SO_4_ 0.5–2.0 g, KH_2_PO_4_ 4.0 g, Na_2_HPO_4_ 0.8 g, NaHCO_3_ 1.0 g, MgSO_4_·7H_2_O 0.2 g and 1 L distilled water. The pH was adjusted to 7.0. After autoclaving, 1.0 mL of filter-sterilized trace elements solution [22] and 200 μg/mL of kanamycin were added to 1 L of the medium. The composition of the trace elements solution was FeCl_3_ 9.7 g, CaCl_2_ 7.8 g, CoCi_2_·6H_2_O 0.218 g, NiCl_3_·6H_2_O 0.118 g, CrCl_3_·6H_2_O 0.105 g and CuSO_4_·6H_2_O 0.156 g per 1 L of 0.1 M HCl. The culture was carried out by using 20 mL of mineral medium in a 300 mL Erlenmeyer flask. The air in the head space within the flask was evacuated by using a vacuum pump after the seed culture was inoculated to the medium in order to set optical density at 600 nm (OD_600_) of the culture broth to 0.1. Then, the substrate gas mixture of the ratio H_2_/O_2_/CO_2_ = 8:1:1 was introduced through a sterile filter. The flask was fitted tightly with a silicon rubber stopper and sealed with adhesive tape (Figure 1). The cells were cultivated at 30 °C, and a reciprocal shaking speed of 170 rpm was used. During the cultivation, the unconsumed substrate gas mixture within the flask was evacuated every 12 h and refilled with new gas mixtures. For each strain and condition, a culture test was carried out in triplicate.

### 2.3. Analyses

Zero point five milliliter of the culture broth was withdrawn from the flask at every 12 h, and the optical density at a wavelength of 600 nm (OD_600_) was measured to monitor cell growth. In order to determine the concentration of dry cell mass (DCM), the cells were harvested by centrifugation after 120 h of cultivation, and the weight of the cells dried at 105 °C was measured. The PHA contents in the cells and monomer composition were determined by gas chromatography. The dry cells were heated in methanol containing 15% sulfuric acid at 100 °C for 140 min for methanolysis of PHA. Then, the methyl esters of 3HB and 3HHx were separated and quantified by gas chromatography [24].

PHA was extracted by stirring the lyophilized cells in chloroform for five days. Then, cell debris was removed by filtration. The filtrate was concentrated with rotary evaporator, and PHA in the condensed extract was precipitated by adding chilled methanol and stirred continuously. The purified PHA was dried in a vacuum at room temperature. Ten milligram of the dried sample was dissolved in 0.7 mL of CDCl_3_ containing 1% TMS, and the polymer solution was applied to 400 MHz ^1^H NMR spectroscopy (Varian 400-MR).

## 3. Result

### 3.1. Autotrophic PHA Synthesis by C. necator MF01/pBPP-ccr_Me_J4a-emd and MF01ΔB1/pBPP-ccr_Me_J4a-emd

The results of flask culture of the engineered *C. necator* strains MF01/pBPP-ccr_Me_J4a-emd and MF01ΔB1/pBPP-ccr_Me_J4a-emd in the autotrophic condition are shown in Table 2. The concentrations of DCM, polymer content in the cells and monomer composition were determined with the samples withdrawn after 120 h of cultivation. It was observed that both recombinant strains vigorously grew and consumed the substrate gasses within the culture flask. When 1.0 g/L (NH_4_)_2_SO_4_ was used as a nitrogen source in the culture medium, DCM of MF01/pBPP-ccr_Me_J4a-emd and MF01ΔB1/pBPP-ccr_Me_J4a-emd increased to 12.18 ± 0.40 g/L and 10.65 ± 1.35 g/L, respectively. At every concentration of (NH_4_)_2_SO_4_, DCM of MF01/pBBP-ccr_Me_J4-emd was higher than that of MF01∆B1/pBBP-ccr_Me_J4a-emd. Contrary to our expectations, DCM in the culture using 2.0 g/L (NH_4_)_2_SO_4_ was inferior to that using 1.0 g/L (NH_4_)_2_SO_4_. In the culture using 2.0 g/L (NH_4_)_2_SO_4_, pH decreased to 4.6, which would inhibit cell growth and PHA accumulation. 

The PHA contents in the recombinant cells and monomer composition determined by GC analysis are also shown in Table 2. It was obvious that these two recombinants synthesized the copolyester of 3HB and 3HHx from CO_2_ in all the cultures tested. The 3HHx composition of PHBHHx produced by MF01∆B1/pBBP-ccr_Me_J4a-emd was much higher than that produced by the corresponding MF01 strain. In particular, 3HHx composition of PHBHHx in MF01∆B1/pBBP-ccr_Me_J4a-emd cultured with 1.0 g/L (NH_4_)_2_SO_4_ medium was 47.7 ± 6.2 mol% (the highest 3HHx composition was 51.7 mol%). On the other hand, the PHBHHx content in the cells was higher in MF01/pBBP-ccr_Me_J4a-emd than in MF01/pBBP-ccr_Me_J4a-emd at any concentration of (NH_4_)_2_SO_4_. The highest PHBHHx content of 85.8 ± 13.2wt% was obtained by MF01/pBPP-ccr_Me_J4a-emd using 0.5 g/L (NH_4_)_2_SO_4_ medium. The lowest value was 21.1 ± 0.5wt% and was obtained by MF01ΔB1/pBPP-ccr_Me_J4a-emd using 2.0 g/L (NH_4_)_2_SO_4_ medium. This tendency of lower PHA content with 2.0 g/L (NH_4_)_2_SO_4_ was also observed for MF01/pBBP-ccr_Me_J4a-emd. It was supposed that, in the cultures using the 2.0 g/L (NH_4_)_2_SO_4_ medium, the decrease in pH impaired cell growth as described above; thus, the concentration of unconsumed NH_4_^+^ in the culture medium was too high to cause nitrogen limitation, which promotes polymer synthesis in cells, resulting in lower polymer contents. 

When the same strains were subjected to heterotrophic culture by using 5.0 g/L fructose as the sole carbon source and 0.2 g/L NH_4_Cl as a nitrogen source, MF01/pBPP-ccr_Me_J4a-emd showed DCM of 1.76 ± 0.02 g/L and accumulation of P(3HB-*co*-6.4 mol% 3HHx) with cellular content of 48.5 wt%, and those by MF01ΔB1/pBPP-ccr_Me_J4a-emd were 1.57 ± 0.02 g/L and P(3HB-*co*-22.2 mol% 3HHx) with 47.9 wt%. Cell growth and PHBHHx synthesis from CO_2_ in the autotrophic condition were better and numbered than those in the heterotrophic condition on fructose.

### 3.2. NMR Analysis of PHBHHx Synthesized by the Autotrophic Condition

**Figure 2:**
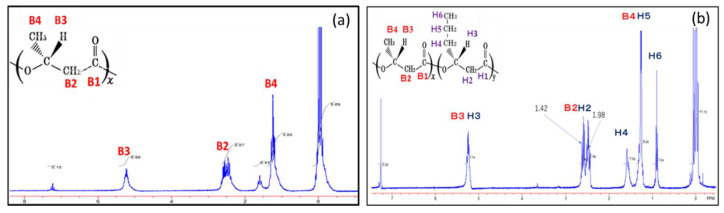
The 400 MHz ^1^H NMR spectrum for P(3HB) synthesized by the wild strain of *C. necator* (**a**) and PHBHHx synthesized by MF01ΔB1/pBPP-ccr_Me_J4a-emd (**b**). Each strain was cultivated in the autotrophic condition with 2.0 g/L (NH_4_)_2_SO_4_.

In the previous study, the structure of PHBHHx synthesized from fructose by the recombinant strains was confirmed by ^1^H- and ^13^C-NMR analyses, and the distribution of 3HB and 3HHx units was statistically random [11]. The copolymer synthesized from CO_2_ was also analyzed by ^1^H-NMR spectroscopy in order to confirm 3HB and 3HHx units in the polymer fraction. The 400 MHz ^1^H-NMR spectra of the polymer synthesized by the wild strain of *C. necator* and that by MF01∆B1/pBBP-ccr_Me_J4a-emd in the culture using 2.0 g/L (NH_4_)_2_SO_4_ medium in the autotrophic condition are shown in Figure 2a,b, respectively. The signals of H4 and H6 observed in Figure 2 were assigned to be resonances of the C4-methylene groups and C6-methyl-group in the 3HHx unit, respectively, indicating actual autotrophic formation of the PHBHHx copolymer. 

### 3.3. Autotrophic PHA Synthesis by C. necator MF01/pBPP-ccr_Me_J_Ac_-emd and MF01ΔB1/pBPP-ccr_Me_J_Ac_-emd

PHBHHx synthesized from CO_2_ by MF01/pBBP-ccr_Me_J4a-emd showed remarkably high 3HHx composition. However, the thermal properties of PHBHHx composed of such very high 3HHx fractions are not good for applications. It is reported that the melting temperature of PHBHHx drastically decreases as 3HHx composition increases [5]. Therefore, we used another plasmid pBPP-ccr_Me_J_Ac_-emd, containing scl-(*R*)-enoyl-CoA hydratase gene *phaJ_A_*_c_ instead of *pha4a*, to repress the excess incorporation of 3HHx unit into the polymer chain. The effects of the substrate specificity of PhaJ on the regulation of 3HHx composition has been confirmed by heterotrophic flask culture using fructose as a carbon source before the autotrophic culture test (Table 3). When MF01ΔB1 was the host strain, the 3HHx composition in PHBHHx synthesized from fructose decreased from 22.2 mol% to 14.0 mol% by the replacement of *phaJ4a* by *phaJ_Ac_* in the plasmid. Other monomers except 3HB and 3HHx were not detected in every recombinant strain.

The results for autotrophic flask culture of the recombinant strains MF01/pBPP-ccr_Me_J_Ac_-emd and MF01ΔB1/pBPP-ccr_Me_J_Ac_-emd at 120 h cultivation are shown in Table 4. The composition of 3HHx in the polymer synthesized by the new strains was successfully controlled in the range from 6.0 ± 1.3 mol% to 14.0 ± 1.3 mol%. In particular, in the culture using the 1.0 g/L (NH_4_)_2_SO_4_ medium, these recombinants synthesized PHBHHx with 3HHx composition around 10 mol%, which is considered to produce physical properties suitable for practical applications.

Compared to PHBHHx biosynthesis from fructose in heterotrophic culture shown in Table 3, the replacement of *phaJ4a* to *phaJ_Ac_* drastically reduced the 3HHx composition in MF01ΔB1 strains in the autotrophic culture. Contrary, in MF01 in which *phaJ4a* was replaced with *phaJ_Ac_*, the 3HHx composition increased in the autotrophic culture.

We tried to continue the flask culture for longer than 120 h with repeated exchange of the substrate gas mixture, aiming to obtain larger DCM. However, the reproducibility of the results of the prolonged culture was very low; in particular, DCM was seriously different even when the same strain was used in the medium with the same composition. 

## 4. Discussion

There have been only a few reports on the biosynthesis of PHA copolymers from CO_2_ by hydrogen-oxidizing bacteria. Volova et al. reported that *Cupriavidus necator* B-10646 produced 50 g/L of DCM with 85% of PHA content in batch culture using a recycled-gas closed-circuit culture system [25]. However, the fraction of other units in addition to 3HB was minor when CO_2_ was used as sole carbon source. The higher fractions of 3HV and 4-hydroxybutyrate and 3HHx monomer units were obtained by supplementing the precursor substrates to the culture medium. According to the report by Heinrich et al., the recombinant of photosynthetic bacterium, *Rhodospirillum rubrum* pBBR1MCS-2::*phaB_Re_*::*pntAB_Ec_*, accumulated PHAs up to 10.1 ± 1.1 wt% of the DCW with 55.5 ± 7.9 mol% of 3HV fraction when an artificial syngas atmosphere composed of 40% CO, 40% H_2_, 10% CO_2_ and 10% N_2_ was used as the carbon and energy sources [26]. Recently, several researchers have reported the biosynthesis of copolymer PHAs with high composition of the second or tertiary unit from CO_2_ as the sole carbon source without using an organic precursor in obligate autotrophic condition (Table 5). Compared to those results, our recombinant strains showed much higher yield and productivity of copolymer PHA from CO_2_.

**Figure 3:**
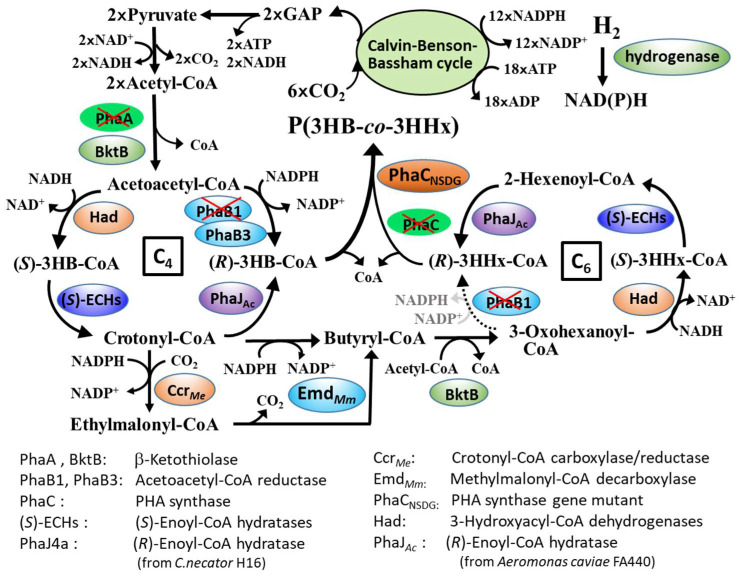
A proposed pathway for PHBHHx biosynthesis from CO_2_ in *C. necator* MF01ΔB1/pBPP-ccr_Me_J_Ac_-emd.

Here, we demonstrated that the engineered strains of *C. necator* capable of synthesizing PHBHHx from sugars can be applicable in copolyester production that is also from CO_2_. Figure 3 shows a proposed pathway for PHBHHx biosynthesis from CO_2_ in the strain MF01ΔB1/pBPP-ccr_Me_J_Ac_-emd. It is known that *C. necator* fixes CO_2_ by the reductive pentose phosphate pathway (Calvin–Benson–Bassham (CBB) cycle) [31]. Under autotrophic conditions, two molecules of glyceraldehyde 3-phosphate (GAP) formed through the CBB cycle were converted to acetoacetyl-CoA and then reduced to (*R*)-3HB-CoA, a C_4_-monomer unit for polymerization. The deletion of *phaB1* weakened the (*R*)-specific reduction of acetoacetyl-CoA, resulting in relative enforcement of competing (*S*)-specific reduction [20]. This was thought to cause efficient formation of crotonyl-CoA via (*S*)-3HB-CoA. Crotonyl-CoA is then converted to butyryl-CoA by combination of Ccr*_Me_* and Emd*_Mm_*, where ethylmalonyl-CoA generated by carboxylase activity of bifunctional Ccr*_Me_* is converted back to butyryl-CoA by Emd*_Mm_*. Alternatively, a part of crotonyl-CoA is used to form (*R*)-3HB-CoA by (*R*)-enoyl-CoA hydratase, PhaJ. Butyryl-CoA acts as a precursor of (*R*)-3HHx-CoA, a C_6_-momomer, and the C_4_-monomers and C_6-_monomers are copolymerized by PhaC_NSDG_. (*R*)-enoyl-CoA hydratase potentially functions in both the interconversion between (*R*)-3HB-CoA and crotonyl-CoA and the formation of (*R*)-3HHx-CoA after the elongation. When *phaJ4a* encoding mcl-specific hydratase was introduced into *C. necator*, the 3HHx composition in the copolyester produced from CO_2_ by MF01ΔB1/pBPP-ccr*_Me_*J4a-emd was surprisingly high (44–48 mol%). This was quite interesting since such high C_6_ compositions have been not observed in heterotrophic biosynthesis, whereas the C_6_-rich copolymer is too soft and, thus, is not suitable for general applications. The copolymer composition could be regulated by adopting another PhaJ in the biosynthesis pathway. It was supposed that scl-specific PhaJ*_Ac_* showing high activity to crotonyl-CoA increases the additional formation of (*R*)-3HB-CoA from crotonyl-CoA accompanied with a relative decrease in the butyryl-CoA formation. As a result, the strain MF01∆B1/pBPP-ccr_Me_J_Ac_-emd produced PHBHHx composed of 14 mol% 3HHx composition with high cellular content on CO_2_.

DCM concentrations in the autotrophic culture of the engineered *C. necator* strains were much higher than those in heterotrophic conditions on fructose. In our previous study, fructose in culture media was limited to 20 g/L because higher fructose concentration was inhibitory to the growth of *C. necator* [11]. In the present autotrophic culture, the substrate gas mixture consumed within the flask was exchanged with a new gas mixture at every 12 h during the cultivation, which enabled attaining much higher DCM concentration than that in heterotrophic culture. In addition, the autotrophic culture also tended to show higher 3HHx composition. Unfortunately, at present, we cannot explain the reason for this phenomenon. It might be that the formation of butyryl-CoA was promoted by the carboxylase activity of Ccr*_Me_* increased by high concentrations of CO_2_ and the activity of Emd*_Mm_*, which increased 3HHx composition in autotrophic conditions. We will investigate the difference in 3HHx composition during heterotrophic culture and autotrophic culture in the future.

Bktb is a medium-chain-length (mcl)-specific β-ketothiolase, which is possible to synthesize various 3-hydroxyacyl-CoAs of different carbon numbers (C_5_–C_10_). However, only C_4_ (3HB) and C_6_ (3HHx) units were detected in the copolymers synthesized from CO_2_ and fructose by GC and ^1^H NMR analyses. In our strains, several enzymes were introduced; however, the genes for synthesis of propionyl-CoA (C_5_) were not introduced. Additionally, 2-hexenoyl-CoA is unlikely transformed to 3-hexanoyl-CoA (that is the precursor of C_8_-CoA) by Ccr*_Me_*/Emd*_Mm_* due to their substrate specificity. PhaC_NSDG_ can hardly polymerize the C_8_ unit. Therefore, it may be that no other units other than 3HB and 3HHx were incorporated in the copolymer.

We already succeeded in high cell density cultivation of wild strain of *C. necator* H16 in an autotrophic culture condition using a 2 L scale jar fermenter and a recycled gas-closed circuit fermentation system. By using specially designed basket type agitation system in the jar fermenter, DCM increased to 91.3 g/L after 40 h of cultivation and homopolyester P(3HB) of 62 g/L was produced from CO_2_, while O_2_ concentrations in the substrate gas mixture within the fermentation system were kept below 6.9% *v*/*v*, which is the lower limit for explosion [15]. We aim to study efficient PHBHHx production from CO_2_ by the *C. necator* recombinants, and we also aim to investigate fermentation characteristics and physical properties of the polymer produced from CO_2_. 

## 5. Conclusions

The biosynthesis of PHBHHx from CO_2_ by engineered strains of a hydrogen oxidizing bacterium *C. necator* was investigated in autotrophic culture using the synthetic mineral medium and substrate gas mixture (H_2_/O_2_/CO_2_ = 8:1:1). The results of GC and ^1^H NMR analyses indicated that the recombinants of *C. necator* synthesized PHBHHx from CO_2_ with high cellular contents in which the maximum value was 85.8 ± 13.2 wt%. The 3HHx composition of PHBHHx in the strain MF01∆B1/pBBP-ccr_Me_J4a-emd when using 1.0 g/L (NH_4_)_2_SO_4_ as a nitrogen source was remarkably high (47.7 ± 6.2 mol%). Further investigation demonstrated that the PHA composition can be regulated by using (*R*)-enoyl-CoA hydratase (PhaJ) with different substrate specificities. The compositions of 3HHx in PHBHHx were controlled to about 11 mol%, which is suitable for practical applications, by keeping high cellular content in the strains transformed with pBPP-ccr_Me_J_Ac_-emd harboring scl-specific PhaJ derived from *A. caviae*. It is expected that the strains transformed with pBPP-ccr_Me_J_Ac_-emd will be useful for the industrialization of PHBHHx production from CO_2_.

## Figures and Tables

**Table 1:** Bacterial strains and plasmids used in this study.

Bacterial Strain or Plasmids	Genotype/Characteristic	References or Sources
*C. necator*		
H16	Wild type	DSM 428
MF01	H16 derivative, *∆phaC*::*phaC*_NSDG_, *∆phaA*::*bktB*	[19]
MF01ΔB1	MF01 derivative; Δ*phaB1*	[11]
Plasmids		
pBPP	pBBR1-MCS2 derivative, *P_phaP1_*, *T_rrnB_*,	[23]
pBPP-ccr_Me_J4a-emd	pBPP derivative, *ccr_Me_*, *phaJ4a*, *emd_Mm_*	[11]
pBPP-ccr_Me_J_Ac_-emd	pBPP derivative, *ccr_Me_*, *phaJ_Ac_*, *emd_Mm_*	[20]

**Table 2:** PHA synthesis by autotrophic flask culture of the engineered *C.* *necator* strains harboring pBPP-ccr_Me_J4a-emd.

Recombinant (Strains/Plasmid Vector)	(NH_4_)_2_SO_4_ (g/L)	DCM (g/L)	PHBHHx Content (wt%)	Monomer Composition (mol%)	
				3HB	3HHx
MF01/pBPP-ccr_Me_J4a-emd	0.5	8.52 ± 1.92	85.8 ± 13.2	96.7 ± 1.4	3.3 ± 1.4
MF01/pBPP-ccr_Me_J4a-emd	1.0	12.18 ± 0.40	64.0 ± 3.4	94.8 ± 1.1	5.3 ± 1.1
MF01/pBPP-ccr_Me_J4a-emd	2.0	8.01 ± 1.65	37.4 ± 2.4	97.7 ± 0.8	2.4 ± 0.8
MF01ΔB1/pBPP-ccr_Me_J4a-emd	0.5	4.35 ± 0.78	59.0 ± 16.2	56.5 ± 1.7	43.5 ± 1.7
MF01ΔB1/pBPP-ccr_Me_J4a-emd	1.0	10.65 ± 1.35	61.7 ± 4.6	52.3 ± 6.2	47.7 ± 6.2
MF01ΔB1/pBPP-ccr_Me_J4a-emd	2.0	6.99 ± 1.14	21.1 ± 0.5	71.6 ± 1.9	28.5 ± 1.9

All data were obtained after 120 h of cultivation (*n* = 3). The substrate gas mixture (H_2_/O_2_/CO_2_ = 8:1:1) in the flask was exchanged every 12 h.

**Table 3:** PHBHHx biosynthesis from fructose by *C. necator* MF01 and MF01ΔB1 expressing different *phaJ* along with *ccr_Me_* and *emd_Mm_*.

Recombinant (Strains/Plasmid)	DCM (g/L)	PHBHHx Content (wt%)	3HHx (mol%)
MF01/pBPP-ccr_Me_J4a-emd ^a^	1.76 ± 0.02	48.5 ± 0.4	6.4 ± 0.13
MF01/pBPP-ccr_Me_J_Ac_-emd	1.81 ± 0.03	54.0 ± 3.1	10.4 ± 0.2
MF01ΔB1/pBPP-ccr_Me_J4a-emd ^a^	1.57 ± 0.02	47.9 ± 2.0	22.2 ± 1.2
MF01ΔB1/pBPP-ccr_Me_J_Ac_-emd	1.72 ± 0.01	54.1 ± 2.1	14.0 ± 0.6

The cells were cultivated in a 100 mL MB medium containing 0.5% (w/v) fructose for 72 h at 30 °C. Standard deviation was shown with each value (*n* = 3). ^a^ Date from previous results [11].

**Table 4:** PHA synthesis by autotrophic flask culture of the engineered *C.* *necator* strains harboring pBPP-ccr_Me_J_Ac_-emd.

Recombinant (Strains/Plasmid Vector)	(NH_4_)_2_ SO_4_ (g/L)	DCM (g/L)	PHBHHx Content (wt%)	Monomer (mol%)	
				3HB	3HHx
MF01/pBPP-ccr_Me_J_Ac_-emd	0.5	7.25 ± 0.57	76.2 ± 0.0	94.0 ± 1.3	6.0 ± 1.3
MF01/pBPP-ccr_Me_J_Ac_-emd	1.0	11.22 ± 2.67	64.6 ± 8.1	88.7 ± 6.4	11.3 ± 6.4
MF01/pBPP-ccr_Me_J_Ac_-emd	2.0	8.46 ± 0.42	19.9 ± 1.7	88.6 ± 0.9	11.5 ± 0.9
MF01ΔB1/pBPP-ccr_Me_J_Ac_-emd	0.5	6.93 ± 0.36	74.6 ± 2.2	86.6 ± 1.0	14.0 ± 1.3
MF01ΔB1/pBPP-ccr_Me_J_Ac_-emd	1.0	8.52 ± 1.00	67.8 ± 1.8	87.1 ± 2.3	11.1 ± 1.3
MF01ΔB1/pBPP-ccr_Me_J_Ac_-emd	2.0	6.03 ± 0.78	39.1 ± 1.3	90.4 ± 0.7	9.6 ± 0.7

All data were obtained after 120 h of cultivation (*n* = 3). The substrate gas mixture (H_2_/O_2_/CO_2_ = 8:1:1) in the flask was exchanged every 12 h.

**Table 5:** Comparison for biosynthesis of copolymer PHAs from CO_2_ as sole carbon source by different autotrophic microorganisms.

Scheme.	Nutritional Condition	PHA Production	PHA Composition (mol%)	Ref.
*C. necator* H 16 ^a^	H_2_/CO_2_/air = 30:15:balance	-	Various type of PHAs with C_4_-C_14_ monomers	[27]
*C. necator* H 16 ^b^	H_2_/O_2_/CO_2_/N_2_ = 3.6: 7.6: 12.3: 76.5 (non-combustible gas)	DCM0.14 ± 0.05 g/L (PHA content 57 ± 10 wt%)	3HB-based copolymer with 1.2 % 3HV ^c^ and 1.2% 3H4MV ^d^	[28]
*Anabaena spiroides* TISTR 8075	CO_2_ + light	PHBV productivity 0.5 mg g_dw_^−1^day^−1^	PHBV (3HV 43.2%)	[29]
*Oscillatoria okeni* TISTR 8549	CO_2_ + light	PHBV concentration 108 mg/L	PHBV (3HV 5.5%)	[30]

^a^ Recombinant expressing different thioesterases and PhaCs; ^b^ Recombinant harboring pBBR1′’C1ABPt_ac_BktB; ^c^ 3HV: 3-hydroxyvalerate; ^d^ 3H4MV: 3-hydroxy-4-methylvalerate.

## Data Availability

Not applicable.
